# Recent Advances and Perspectives on the Sources and Detection of Antibiotics in Aquatic Environments

**DOI:** 10.1155/2022/5091181

**Published:** 2022-05-25

**Authors:** Yanbo Zeng, Fengqin Chang, Qi Liu, Lizeng Duan, Donglin Li, Hucai Zhang

**Affiliations:** Institute for Ecological Research and Pollution Control of Plateau Lakes, School of Ecology and Environmental Science, Yunnan University, Kunming, Yunnan 650504, China

## Abstract

Water quality and safety are vital to the ecological environment, social development, and ecological susceptibility. The extensive use and continuous discharge of antibiotics have caused serious water pollution; antibiotics are widely found in freshwater, drinking water, and reservoirs; and this pollution has become a common phenomenon and challenge in global water ecosystems, as water polluted by antibiotics poses serious risks to human health and the ecological environment. Therefore, the antibiotic content in water should be identified, monitored, and eliminated. Nevertheless, there is no single method that can detect all different types of antibiotics, so various techniques are often combined to produce reliable results. This review summarizes the sources of antibiotic pollution in water, covering three main aspects: (1) wastewater discharges from domestic sewage, (2) medical wastewater, and (3) animal physiology and aquaculture. The existing analytical techniques, including extraction techniques, conventional detection methods, and biosensors, are reviewed. The electrochemical biosensors have become a research hotspot in recent years because of their rapid detection, high efficiency, and portability, and the use of nanoparticles contributes to these outstanding qualities. Additionally, the comprehensive quality evaluation of various detection methods, including the linear detection range, detection limit (LOD), and recovery rate, is discussed, and the future of this research field is also prospected.

## 1. Introduction

Antibiotics refer to a class of secondary metabolites with antipathogenic or other activities produced by microorganisms or higher animals and plants during their lives; these chemical substances can interfere with the development of other cells and are widely used in human and veterinary medicine [1], agricultural production, animal husbandry, aquaculture, and other fields [2, 3]. With the high production and consumption of antibiotics in recent years, some medicines and personal care products, including antibiotics, antibacterial agents, and other emerging pollutants, have entered water bodies [4, 5], and the rest are spread into the soil through manure and sludge [6] and migrate between the water phase and the solid phase.

Antibiotics make bacteria resistant and pose an unpredictable risk to human health and safety [7]; these compounds enter the environment and affect ecosystem functions because they can kill microorganisms or inhibit their growth and are likely to disrupt the balance of microorganisms in the ecosystem [8], leading to the reduction of biodiversity and degradation of ecosystems [9, 10]. Accordingly, to help treat infectious diseases, antibiotics are used in large quantities, and the content of antibiotics in wastewater is increasing rapidly. Moreover, conventional sewage treatment plants cannot effectively degrade antibiotics [11], which objectively impacts the ecological environment.

**Figure 1:**
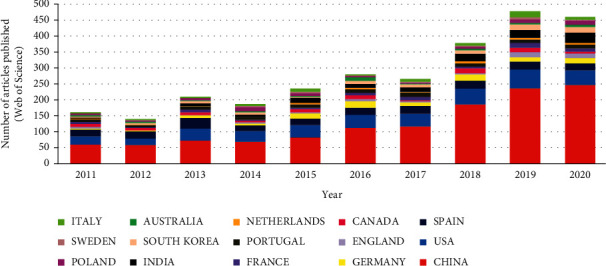
Research publications on antibiotic detection in water in the last decade (2011–2020). Source: Web of Science (https://www.webofknowledge.com).

For these reasons, it is important to detect antibiotics in water accurately and assess their impact on human health and the ecological environment [12]. In recent years, there have been many studies on the detection of antibiotics in water.  Figure 1 presents the number of research papers and scientific reports related to the detection and quantification of antibiotics in water over the past 10 years (2011–2020), showing an increasing trend year by year. The data shown in Figure 1 were indexed on the Web of Science using the keyword “detection of antibiotics in water,” and then the results were classified using the filters “year” and “country/region.”

Many reviews focus on the electrochemical, optical, and biosensor detection of antibiotics; however, there has been no comprehensive review on the sources of antibiotic contamination and various detection methods. In this paper, we reviewed several recent studies about the sources and detection technology of antibiotic contamination in waterways and the development trend of antibiotic detection prospects.

## 2. Sources of Antibiotic Pollution in the Watershed

Antibiotics are disseminated into the water environment in three main ways: (1) medical wastewater [13, 14], (2) discharges from domestic sewage [12], and (3) animal husbandry and aquaculture [15–17].

### 2.1. Medical Wastewater

The industrial pollution of antibiotics mainly comes from the production processes of antibiotic manufacturing enterprises, including residues of antibiotics in waste residue, production of wastewater after treatment, and residual sludge. Additionally, the treatment and disposal of expired drugs cause environmental pollution. The industrial production of antibiotics produces a large amount of wastewater, and this wastewater often contains a high concentration of antibiotics. In some countries, the concentration of antibiotics in wastewater discharged by antibiotic manufacturing enterprises can reach the ppm level [18], and in sewage treatment plants in some countries, antibiotics from antibiotic manufacturing plants also contribute significantly to the total antibiotic concentration. Hospital sewage is another important source of antibiotic residue pollution. For example, a positive correlation was observed between the amount of ciprofloxacin prescribed in hospitals and antibiotic residue levels in hospital effluents [19, 20].

Since antibiotics have not been included in the current wastewater discharge standards, the effluent and residual sludge of antibiotic wastewater releases a variety of potentially high-risk antibiotics to the natural environment, including drug transformation intermediates, endocrine disruptors, and antibiotics [21]. Moreover, some antibiotics cannot be completely removed from the waste residue generated during the production and treatment of antibiotics and the disposal of expired drugs, which also causes residual antibiotics to enter the water environment [12]. Therefore, antibiotic manufacturers and hospital sewage are an important source of antibiotic pollution in the environment.

### 2.2. Discharges from Domestic Sewage

Another important source of antibiotic pollution in the environment is domestic sewage discharge. Studies have shown that only a small portion of antibiotics entering the organic body is absorbed and utilized by organisms, and 50%∼80% of antibiotics cannot be completely absorbed but will be discharged in the form of active drugs or metabolites through feces and urine and enter sewage treatment facilities [22]; the reason is that the existing wastewater treatment processes in sewage treatment plants cannot completely degrade antibiotics. Residual antibiotics eventually enter the environment with the drainage of sewage treatment plants and are considered to be an important source of antibiotics and other drugs and their secondary metabolites in the water environment [15]. Due to the different types and amounts of antibiotics used in different countries and regions, there are also major differences in antibiotic-containing wastewater treatment, resulting in significant differences in the types and concentrations of antibiotics in the effluents of different sewage treatment plants.

Any antibiotic remaining in the effluent from any sewage treatment plant is eventually discharged into the water; moreover, human and animal waste are input into farmland in the form of organic manure, and the residual sludge from the sewage treatment process will eventually enter the soil environment and enter groundwater, surface water, and sediments through different migration pathways [23]. Improper disposal of unused or expired antibiotic medicines, such as those thrown into toilets or the garbage, is also seen as an important potential source of pollution in the water environment.

### 2.3. Animal Husbandry and Aquaculture

In the process of large-scale animal husbandry, antibiotics are not only used to prevent and treat animal infectious diseases but also added to feed as growth promoters [24], resulting in a large number of antibiotics in aquaculture wastewater and animal feces, which enter the water, sediments, and soil through point or nonpoint source pollution.

Additionally, a large number of antibiotics are used in aquaculture. Due to the low utilization rate of aquaculture drugs, only 20%∼30% can be absorbed by aquaculture products. The remaining large amount of antibiotics is released into the surrounding water body or the sediment of the aquaculture area through nonpoint source pollution and continues to spread and migrate.

In the past, Chinese aquaculture was concentrated in the Taihu Lake basin where veterinary antibiotics were found at high concentrations [25, 26]. Globally, the production and use of antibiotics are increasing greatly. Incomplete removal of antibiotics in urban sewage treatment plants leads to a large amount of them continuously entering the water environment. As shown in Table 1, antibiotics have been detected in the water bodies of some countries. The uncontrolled use of antibiotics and their discharge into the environment lead to increase in bacterial resistance, which makes it difficult to control diseases and appearance of highly resistant strains [27]; hence, the control of antibiotics is vital to the protection of human health and environment safety.

## 3. Extraction Techniques

### 3.1. Liquid-Liquid Extraction (LLE)

**Figure 2:**
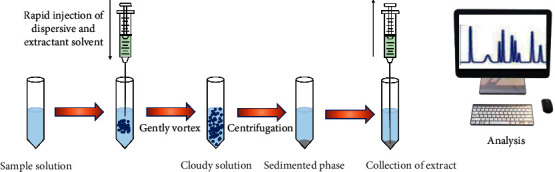
Schematic procedure of the dispersive liquid-liquid microextraction technique (the image is from [39]).

Liquid-liquid extraction is the process of transferring organic matter from one solvent to another by taking advantage of the different solubilities of organic matter in two immiscible solvents. As shown in Figure 2, dispersive liquid-liquid microextraction (DLLME) has high extraction efficiency, stability, and sensitivity and a simple sample pretreatment procedure. In recent years, this technique has been widely used in the enrichment and determination of compounds. For example, in 2016, Razmi et al. [36] established a method for rapid extraction and enrichment of ceftazidime by DLLME, and this method has been successfully used for the determination of ceftazidime in aqueous samples.

Most of the reported microextraction methods focus on the extraction of compounds with similar properties. To meet the technical requirements of the simultaneous determination of environmental pollutants with large polarity differences, a number of new methods have been developed. In 2017, Gao et al. [37] developed a method called salting-out-enhanced ionic liquid microextraction based on a dual-role solvent (SILM-DS). The dispersant in the ionic-liquid-based dispersion-liquid-liquid microextraction is converted to the extraction solvent in the subsequent salting-out-assisted microextraction process so that the single solvent has the dual function of extractant and dispersant in the SILM-DS process. This technology combines the advantages of dispersion-liquid microextraction (DLM) and salting-out-assisted microextraction to achieve higher extraction efficiency for drugs and phenolic contaminants with a wide range of polarities in aqueous samples. This method can meet the technical requirements of simultaneously determining environmental pollutants with large polarity differences and can simultaneously and quantitatively analyze a variety of antibiotic residues in complex milk and environmental water substrates. This approach has great application potential for the simultaneous determination of trace pollutants with strong polarity contrast in multiple analytical fields.

### 3.2. Dispersive Liquid-Liquid Microextraction

To further improve the efficiency of liquid-liquid extraction, In 2016, Guan et al. [38] adopted an ultrasonic-assisted process to accelerate the formation of a fine-turbidity solution by dispersing the solvent in a small volume so that the organic solvent was dispersed into droplets, and both the aqueous phase and the organic phase had a considerable contact area, thereby improving the extraction efficiency and reducing the equilibrium time.

### 3.3. Solid-Phase Extraction (SPE)

The use of liquid-liquid extraction is rare for three main reasons: (1) time-consuming extraction period, (2) cumbersome procedure and use of expensive glassware, and (3) large quantity of extraction solvent needed. Therefore, SPE is a more frequently used extraction technique.

Solid-phase extraction means that the target compound in the liquid sample is adsorbed by the solid adsorbent, separated from the matrix and interfering compound of the sample, and then eluted or desorbed by elution liquid or heating to separate and concentrate the target compound.

### 3.4. Magnetic Solid-Phase Extraction (MSPE)

Magnetic solid-phase extraction has been widely used to enrich a variety of compounds, including metal ions [40], organic compounds [41], and biomolecules [42]. Magnetic solid-phase extraction (MSPE) using magnetic nanomaterials is considered to be an interesting technique for the analysis of contaminants in liquid samples. After they are dispersed into solution, magnetic nanoparticles (MNPs) containing the analyte can be easily separated from the sample solution using magnets, and the compound can then be eluted from the adsorbent with appropriate organic solvents. Using this principle, Pérez [43] and colleagues performed magnetic solid-phase extraction of tilmicosin, erythromycin, tylosin, and erythromycin-H_2_O from water samples using functionalized magnetic nanoparticles.

Similarly, in 2019, Tang et al. [44] used graphene oxide-functionalized magnetic composites (GO@NH_2_@Fe_3_O_4_) as magnetic SPE adsorbents to establish a simple and highly sensitive method for screening 12 kinds of quinolone antibiotics. The method has a high affinity for quinolones and was suggested to be beneficial to the detection of antibiotics in water samples. In 2019, Xiao [45] and colleagues proposed a simple strategy to construct a three-dimensional porous structure of an MSPE-HPLC adsorbent and established a sensitive and effective MSPE-HPLC analysis method for the analysis of antibiotics in water. The prepared Fe_3_O_4_/Go/MoS_2_ magnetic nanocomposites exhibited good dispersion in aqueous solution and good enrichment ability for fluoroquinolone antibiotics, and the analytes were adsorbed within a short time of approximately 2 min. This method is simple, rapid, and sensitive and has potential application value in the detection of antibiotics in polluted water.

### 3.5. Molecular Imprinted Polymers (MIPs)

With the wide application of antibiotics in livestock, increasing attention has been given to pollution of the aquatic environment. To overcome the disadvantages of traditional solid-phase extraction, such as poor column selectivity, poor durability, and low number of repeated uses, molecularly imprinted polymers, as a kind of environmentally friendly, durable, and high-specificity solid-phase extraction material, have shown great potential for avoiding water quality issues during separation and enriching ultratrace amounts of target antibiotic compounds in water. To detect multiple antibiotics in water samples, in 2018, Song et al. [46] established a method based on molecularly imprinted solid-phase extraction (MISPE) for simultaneous enrichment of 10 macrolides in water samples from different sources. The MIP had a good carrying capacity in water (>90 mL), which was more than 3 times that of a non-molecularly imprinted polymer (NIP).

### 3.6. Solid-Phase Extraction in Combination with Dispersive Liquid-Liquid Microextraction

Considering the advantages of SPE and DLLME, it is worthwhile to combine them. Liang [47] and others established a new method of SPE-DLLME for super-enrichment of 10 antibiotics in water samples from different environments. This combined approach can not only enrich ultratrace antibiotics but also effectively reduce the matrix effect.

## 4. Conventional Detection Methods

### 4.1. Detection Methods Based on Liquid Chromatography

As liquid chromatography is not affected by the characteristics of poor volatility and thermal stability of antibiotics, combined chromatographic techniques are widely used in the detection of antibiotics in different media such as fresh water [48], sea water [49, 50], drinking water [51], sediment [52, 53], and food [54, 55]. Liquid chromatography has become the most widely used method in the detection of antibiotic residues, with the advantages of easy operation and fast analysis speed.

Antibiotics are usually separated using high-performance liquid chromatography [56], which provides qualitative and quantitative information through different detection systems [57]. Chromatography or its combination with a variety of quantitative detectors is the commonly used antibiotic detection method. These methods are preferred because they can meet different detection accuracy requirements. Common detection methods include ultraviolet-visible (UV) spectroscopy, fluorescence detection (FD), tandem mass spectrometry (MS/MS), and other combined technologies.

### 4.2. Liquid Chromatography-Ultraviolet

For the detection and analysis of pollutants in water, high-performance liquid chromatography is generally combined with UV-visible detectors to detect ultraviolet absorbance.

Xiao [45] and others used MoS_2_-graphene oxide magnetic nanoparticles as adsorbents to analyze antibiotics in water, including levofloxacin, pazufloxacin, and gatifloxacin, by HPLC. A sensitive and effective HPLC tandem UV detection method was established with a detection limit of 0.25∼0.50 ng·mL^−1^, the recoveries of water samples ranged from 85.6% to 106.1%, and the relative standard deviation (RSD, *n* = 5) was less than 9.5%. A similar approach was applied by Turiel et al. [58], who used a high-performance liquid chromatography (HPLC) tandem UV detection method to analyze multiple residues of several quinolones and fluoroquinolones in surface water; first, preconcentration was performed by SPE, and then quantification was performed by liquid chromatography-tandem UV detection. The detection limit was between 8 and 20 ng/L, proving that this approach is suitable for the determination of (fluorine) quinolones in water samples at realistic ambient concentration levels.

### 4.3. Liquid Chromatography-Fluorescence

Ultraviolet detectors have a wide range of uses, but their sensitivity is limited. For some high-fluorescence analytes, liquid chromatography with fluorescence detection can be applied for analysis. This combined technique has a relatively high sensitivity and can determine low concentration analytes.

Golet [59] and colleagues reported a specific and sensitive analytical method based on the reversed-phase liquid chromatograph-fluorescence detection (LC-FLD) analysis method, and by recording the fluorescence spectrum and liquid chromatography-tandem mass spectrometry, direct coupling to FQs in urban sewage was determined. At the same time, the method was proved by simultaneous determination of nine FQs and quinolone pipemidic acid in urban wastewater, which was successfully applied in FQ quantification of urban sewage treatment plant effluent. In 2018, Yang [60] and others established an ultraperformance liquid chromatography-fluorescence method for the determination of sulfonamides in water. The detection of target sulfonamides was linear in the concentration range of 0.05–5 *μ*g/L, and the correlation coefficient was 0.9924–0.9994. The limits of detection and limits of quantification of 8 sulfonamides were 3.1∼11.2 ng/L and 10.3∼37.3 ng/L, respectively. The enrichment coefficients of 0.1 and 5 *μ*g/L sulfonamides in lake water ranged from 14 to 60, and the recoveries ranged from 56% to 113% with relative standard deviations (RSDs) of 3% to 19%. Yiruhan et al. [51] used high-performance liquid chromatography to detect fluoroquinolone antibiotics (norfloxacin, ciprofloxacin, lomefloxacin, enrofloxacin) in tap water from Guangzhou and Macao. The limit of detection was 1.0∼679.7 ng/L (SD ≤ 37.6) in Guangzhou and 2.0∼37.0 ng/L (SD ≤ 2.5) in Macao. The results show widespread contamination of the tap water of Guangzhou and Macau by fluoroquinolone antibiotics.

### 4.4. Liquid Chromatography-Mass Spectrometry

Liquid chromatography-tandem mass spectrometry is the most commonly used method for the detection of antibiotics because of its high sensitivity, high accuracy, and high selectivity [61, 62].

To enrich ultratrace antibiotics and effectively reduce matrix effects, Liang [47] and colleagues established a new method for the detection of 10 antibiotics in environmental water samples by solid-phase extraction-dispersion-liquid microextraction combined with ultraperformance liquid chromatography-tandem mass spectrometry. The LOD and LOQ of this method were less than 1.67 and 5.57 ng·mL^−1^, respectively; the relative recoveries were between 64.16% and 99.80%; and the relative standard deviations (RSDs) were between 0.7% and 8.4%. This method has been successfully applied to the extraction and analysis of antibiotics in different water samples. In the same way, in 2020, Goessens [63] and others used UPLC-MS to detect sulfonamides, tetracyclines, and other antibiotics by full scanning; all the compounds showed a good linear relationship (*R*^2^ ≥ 0.993), the goodness-of-fit coefficient (*G*) was ≤11.56%, the limit of detection was 10–50 ng·L^−1^, and the limit of quantification was 50 ng·L^−1^. A total of 20 tetracycline antibiotics, 3 antimicrobial degradation products, and 11 species were detected from 18 freshwater ponds throughout Flanders. Pérez et al. [43] performed magnetic solid-phase extraction using oleic acid-functionalized magnetic nanoparticles, optimized various experimental parameters that affected extraction efficiency, and then performed LC-MS/MS analysis. The recoveries of all compounds, except erythromycin, were BBB 0.84%. The LOD values ranged from 11.5 to 26 ng·L^−1^, and the LOQ values ranged from 34 to 77 ng·L^−1^. The method has been applied to the monitoring of pollutants in water samples from different sources. Song [46] et al. established a method based on molecularly imprinted solid-phase extraction (MISPE) combined with liquid chromatography-tandem mass spectrometry (LC-MS/MS) for the simultaneous detection of 10 macrolides in water samples from different sources because MIPs have a good carrying capacity for water (>90 mL), which is more than three times that of non-molecularly imprinted polymer (NIP). The average recoveries of macrolides at four spiked concentrations (limits of quantification: 40, 100, and 400 ng/L) ranged from 62.6 to 100.9%, with intraday and interday relative standard deviations (RSDs) less than 12.6%. The limits of detection were 1.0∼15.0 ng/L, and the limits of quantification were 3.0∼40.0 ng/L. Finally, the method was successfully applied to the analysis of real water samples. In 2020, Duan et al. [64] determined 10 kinds of sulfonamides (SAs) in tap water, lake water, and river water by using solid-phase microextraction combined with UPLC-MS/MS analysis; the limit of quantification of SAs was 0.54∼4.5 ng·L^−1^ using only 1 mL of the water sample. The recoveries were 82.0%∼105.4%, and the intraday and interday relative standard deviations were 3.3%–5.6% and 4.2%–8.1%, respectively. This method requires fewer samples than other methods. The results show that this method can selectively separate and enrich trace nitrogen-containing SAs in water.

To save detection time, in 2019, Huang et al. [65] established a method for the identification and quantification of 18 kinds of antibiotics in urine by liquid chromatogram-triple quadrupole tandem mass spectrometry (LC-QQQ-MS/MS). All the compounds were separated and detected within 15 min, and the limit of quantification for most of the target compounds was 0.3∼7.5 *μ*g/L. The recoveries of all antibiotics were 73%∼136%, except for macrolides whose recoveries were 52%∼78%, and higher accuracy was obtained. The method can quantify antibiotics in urine for monitoring antibiotic consumption or pharmacokinetic studies. To complete the test more efficiently, in 2015, Xue et al. [66] adjusted the pH and eluent and optimized the solid-phase extraction conditions to establish an ultrahigh-performance liquid chromatography-electrospray tandem mass spectrometry (UPLC/MS/MS) method, which was applied to the analysis of 35 kinds of antibiotics including sulfa drugs (SAs), quinolones (QLs), tetracyclines (TCs), macrolides (MALs), clindamycin (LIN), and chloramphenicol (CAP). The detection was completed within 10 min, and the method detection limit (MDL) was 0.29∼4.03 ng/L. The recoveries of antibiotics in ultrapure water and groundwater were 67.13–93.00% and 68.91–92.67%, respectively. The detection of antibiotics in wastewater, surface water, and groundwater samples was also effective, and the method was successfully used for water sample analysis of livestock wastewater, surface water, and groundwater in Tianjin and Beijing, China.  Table 2 summarizes the liquid chromatography-based conventional detection methods for the detection of antibiotics.

## 5. Biosensors

### 5.1. Optical Biosensors

Although a variety of chromatography-based analytical techniques can be used to detect antibiotics [71], the existing equipment is too expensive, and sample pretreatment is time-consuming and laborious [72–76]. Therefore, it is more practical to develop an effective method to detect antibiotics in water environments quickly and sensitively. The principle of the optical sensor is to capture the signal generated by the interaction between a biometric element and the target analyte and convert it into an optical signal; this approach is widely used for antibiotic detection due to its simplicity, convenience, and sensitivity [77]. Based on the mechanism of optical signal transduction, fluorescence and colorimetry techniques are reviewed in this paper.

#### 5.1.1. Colorimetric Biosensors

The colorimetric method has aroused great interest because of its simple preparation, rapid detection, and visible color changes. Given these properties, colorimetric biosensors are very popular in the detection of antibiotics in water.

Because tetracyclines (TCs) strongly bind to metal ions such as Fe(II) and Fe(III) on the surface of Fe_3_O_4_ MNPs, in 2016, Wang [78] et al. developed a tetracycline colorimetric biosensor based on Fe_3_O_4_ magnetic nanoparticles (Fe_3_O_4_ MNPs), where the complexation of Fe_3_O_4_ MNPs with TCs can accelerate the oxidation of 3,3′,5,5′-tetramethylbenzidine (TMB) by H_2_O_2_ to make the solution dark. The analysis results can be observed by the naked eye and then detected by UV-Vis spectroscopy. Under the optimal conditions, the limits of detection of oxytetracycline (OTC), tetracycline (TC), and doxycycline (DOX) were reported to be 26 nM, 45 nM, and 48 nM, respectively. Based on this work, in 2018, Wang et al. [79] designed a new fluorometric method based on carbon quantum dots (CDs), using CDs as a fluorescence probe to detect oxytetracycline (OTC) in the presence of Fe_3_O_4_ MNPs and H_2_O_2_. OH can quench the fluorescence of CDs faster and more effectively. Meanwhile, the absorption band of OTC and the excitation band of CDs have complementary overlapping regions, resulting in the fluorescence quenching of CDs by the inner filter effect between OTC and CDs. Thus, a fluorescence sensor for OTC detection was constructed with a detection limit of 9.5 nM. The colorimetric biosensor is easy to operate and requires no complex chemical synthesis, modification, or tedious experimental process. Signal generation and detection are simple and can be completed by the naked eye. Similarly, by combining nanomaterials with specific recognition biomolecules based on the peroxidase activity of gold nanoparticles (AuNPs) and their interaction with chain peptide complexes, Zhao et al. [80] developed a new type of colorimetric adapter body sensor for streptomycin (STR) detection; by optimization of the reactant concentration and pH, under the best conditions, the sensor could detect STR in the range 0.1∼0.5 mM, with a detection limit of 86 nM, and could be applied to the analysis of STR in milk samples.

#### 5.1.2. Fluorescent Biosensors

Fluorescence-based chemical sensors have attracted wide attention because of their fast response, high sensitivity, and simple preparation [81–83].

In 2012, Song et al. [84] established a dual fluorescence colorimetric method based on gold nanoparticles as an aptamer sensor to detect ampicillin, enabling observation of fluorescence differences and color changes. The detection limits of this sensor are all lower than the maximum residue limit of ampicillin, proving the remarkable sensitivity of this dual detection method. This approach has been shown to be an accurate test for antibiotics in foods such as milk. Yuan et al. [76] proposed a method for detecting oxytetracycline (OTC) using an aptamer probe based on indirect fluorescent labeling. The method showed good sensitivity and selectivity through combination of the target OTC with its aptamer. The linear response range of this method was 0.01∼0.2 mM, and the limit of quantification was 0.01 mM. Through the recovery experiment of the labeled samples, the feasibility of this method in the detection of pollutants in fresh water was verified. This achievement not only realizes the detection of OTC but also lays the foundation for quantitative analysis of small molecules in the environment by an indirect labeling method.

To avoid tedious modification of the electrode, in 2016, Tan et al. [85] first established a fluorescence assay of GO hydrogels based on the target response for the detection of antibiotics; by physically mixing GO, adenosine, and the adaptive system to prepare hydrogels, immobilization and/or modification processes were avoided, and quantitative OTC detection was achieved under mild conditions. Compared with the previously described method, the hydrogel sensor has certain thermal and chemical stability. The linear response to oxytetracycline (OTC) is in the range of 25∼1000 *μ*g/L, and the limit of quantification is 25 *μ*g/L. The authors also tested hydrogels designed to replace recognition elements in order to detect other molecules in general. Based on the principle that a variety of fluoroquinolones can selectively quench the fluorescence of MOFs (in suspension), in 2020, Zhong et al. [86] obtained a detection limit close to 50 ppb for fluoroquinolones. To further broaden the pH range of detection, Zhu et al. [87] reported the use of a Zn(II)-based fluorescent metal-organic framework (MOF) material to detect sulfonamide antibiotics in the aqueous phase based on the fluorescence quenching effect. This method has a wide application range of pH (3.0∼9.0) and is not affected by the presence of heavy metal ions, thereby showing broad application prospects in wastewater treatment and water quality monitoring. In 2021, Wang et al. [88] synthesized a new 3D cluster-based MOF, [Eu_2_(dtztp) (OH)_2_(DMF) (H_2_O)_2.5_] 2H_2_O, by solvothermal method. The MOF showed strong fluorescence; importantly, it can quickly detect metronidazole (MDZ) and dimetridazole (DMZ) antibiotics in water (pH = 3–12), with good recovery rate and low detection limit.

### 5.2. Electrochemical Biosensors

**Figure 3:**
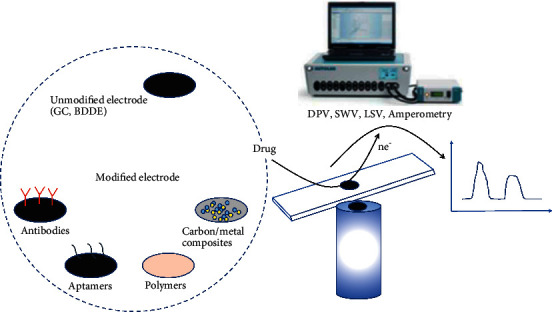
Several modification strategies and electrochemical techniques for drug analysis (the image is from [93]).

Electrochemical biosensors convert analyte concentrations into electrical signals for measurement purposes; they have significant advantages over other biosensors due to their high sensitivity and ability to sense samples with high turbidity and are not affected by interference from absorbance and fluorescent compounds [89]. Nanomaterials, due to their unique structure, can provide attachment sites for bioactive components, facilitating their immobilization for use. In addition, the materials themselves have high catalytic activity, electrical conductivity, and biocompatibility, exhibiting reduced overpotential that can accelerate signal transduction and amplify the signal, resulting in highly sensitive and specific biosensing detection. Furthermore, comprehensive analysis of past literature confirms that smart nanomaterials with the coupling of electrochemical methods significantly improve the analytical performance of the sensors [90–92].  Figure 3 shows the electrode modification strategy for the electrochemical detection of drugs.

Many functionalized nanocomposites can amplify electrochemical signals, for sensitive and quick detection of *Salmonella*, in 2020, Huang [94] et al. developed ZnO-capped mesoporous silica nanoparticles for signal amplification. In 1.5 hours, the biosensor was able to detect *S.* Typhimurium at concentrations ranging from 102 to 107 CFU/mL. To increase electrochemical signals for *Salmonella* Typhimurium detection, an electrochemical immunosensor based on ferrocene (Fc)-functionalized nanocomposites was constructed. The ferrocene (Fc)-functionalized nanocomposites can amplify the electrochemical signals, with excellent specificity and accuracy. The immunosensor platform has a low limit of detection (LOD) of 3 CFU·mL^−1^ and a linear range of 10 to 10^7^ CFU·mL^−1^ in 2020 [95]. The effect of individual nanoparticles is very limited, as graphene derivatives and carbon nanomaterials, because they have a high surface/volume ratio with a large number of active adsorption sites, can improve electrical and catalytic properties and increase sensitivity, using the synergistic effect of metal and carbon-based nanomaterials, which not only effectively prevents agglomeration but also confers high electrode interface electronic conductivity and improved electron transfer kinetics by increasing the electroactive surface area of the interface, significantly improving current response and detection performance. In 2019, Sebastian et al. [96] described how they used the benefits of GO with nanosheet-based hierarchical ZnO to create a sensitive electrochemical sensor for chloramphenicol. An aqueous solution approach was used to create a three-dimensional flower-like ZnO structure made up of nanosheet subunits, which was then composited with GO using a simple sonochemical process. The GO/ZnO nanocomposite was employed to alter a glassy carbon electrode (GCE) and tested for CAP detection. The CAP detection limit of the GO/ZnO/GCE is 0.01 *μ*M, with a high sensitivity of 7.27 *μ*A·*μ*M^−1^·cm^−2^ and a linear response in the 0.2 to 7.2 *μ*M range. In 2021, Ramadhass et al. [97] synthesized cerium (Ce)-doped tungsten oxide/graphene oxide (CeW/GO) composites by hydrothermal method and conducted electrochemical detection of furazolidone (FUZ) by voltammetry. Under the optimal conditions, the detection limit is 0.054 *μ*M, the sensitivity is 2.5 *μ*A·*μ*M^−1^·cm^−2^, and the linear range is 1–260 *μ*M. The sensor was successfully used to detect FUZ in human urine. In 2018, Li et al. [98] designed a three-probe electrochemical sensing sensor based on a free signal probe for the detection of kanamycin A. The sensor overcomes the shortcoming of the close distance between the redox-modified probe and the electrode surface, inhibits the background signal, and improves the detection sensitivity. The detection limit of the sensor for kanamycin A is 3.3 pM, and the response time is 9 min. Based on a previous report of a signaling-probe displacement electrochemical APTA sensor for kanamycin A, Yang et al. [99] developed a second-generation sensor by optimizing the surface chemistry. The sensor has excellent anti-interference capability, with detection limits of 0.28 nM and 7 nM for AMPI and sulfadimethoxine (SDM) in lake water, respectively. The device can be used for the detection of lipophilic compounds in water.

Jacobs et al. [118] developed an electrochemical sensor for detecting trace amounts of erythromycin. This sensor is capable of detecting erythromycin with a sensitivity and specificity of 0.001 parts per trillion in various water samples and has potential applications in environmental water quality assessment. In 2017, Feier et al. [119] assessed the electrochemical oxidation of seven cephalosporins (ceftriaxone, cefotaxime, ceftazidime, cefadroxil, cefuroxime, cefaclor, and cefalexin) at high potential using a bare boron-doped diamond electrode, and the influence on the analytical response of the side chains was investigated. Based on the anodic oxidation of the cephalosporin nucleus, a simple and sensitive method was developed for the electrochemical detection of cefalexin by differential pulse voltammetry. After optimization of the experimental conditions, a linear correlation was obtained between the peak height and the molar concentration of cefalexin in the range of 0.5 *µ*M–700 *µ*M, with a limit of detection of 34.74 ng·mL^−1^. For this method, the peak of cefalexin (CFX) decreases in the presence of other cephalosporins, and the selectivity of this method needs to be improved. To achieve this goal, in 2019, Feier et al. [120] developed an electrochemical sensor based on MIP for sensitivity and selectivity detection of CFX. The electrode showed a linear response to CFX concentrations ranging from 10 to 1000 nM, and the detection limits of the boron-doped diamond electrode and glassy carbon electrode were 3.2 nM and 4.9 nM, respectively. This method has been successfully applied to the determination of cefalexin in the actual environment and drug samples.  Table 3 summarizes the optical/electrochemical biosensors for the detection of antibiotics; regarding the detection of antibiotics in water, the characteristics of the conventional detection method and the biosensor method are listed in Table 4.

## 6. Conclusion and Prospects

In this paper, the sources, advantages, and disadvantages of the detection methods of antibiotic pollution in water environment in recent years were reviewed and discussed.

In general, the conventional detection method is accurate with high sensitivity and selectivity, but its drawbacks and disadvantages are also obvious, for example, the expensive equipment, time-consuming pretreatment, and necessity for professional operators. Biosensor methods offer more important advances in the detection of antibiotics in water, though each type of biosensor has its own advantages and disadvantages, and in comparison, electrochemical biosensors offer significant advantages. Nanomaterial-based electrochemical biosensors for antibiotic detection have been widely used, demonstrating low operating potentials, low detection limits at the nanomolar level, and wide linear analytical ranges without interference for antibiotics. The application of different types of nanomaterials can impart high conductivity to the electrode interface and also increase the electroactive surface area of the interface, thus significantly improving the current response and detection performance. The successful application of nanomaterial-based electrochemical biosensors for the analysis of antibiotics in various matrices demonstrates their reliability as portable field analytical sensors and is a promising technology in antibiotic detection methods.

Even though biosensors have shown significant advances, there are still issues that need to be solved, such as adapting the design of sensing platforms to explore the full potential of sensors, immobilization of different bioreceptors used to construct array systems, ensuring biocompatibility of materials and biometric molecules, effect of nanoparticle inhomogeneity on biosensor performance, cross-interference within antibiotics, and effective linkages between nanomaterials and biorecognition molecules. We still need a robust technology capable of generating large quantities of high-quality, high-precision, small-size, portable, and low-cost nanomaterial-based biosensors urgently. Future efforts should focus on improving biorecognition elements to enhance their stability under real environmental conditions. The binding activity of almost all types of biorecognition components can be affected by sample variables such as temperature, pH, viscosity, and ionic strength. Nonspecific interactions of interfering compounds present in bioreceptors are also a significant challenge, and finally integration of electrochemical sensors with other types of sensors like optical sensors or technologies including microfluidics and the Internet of Things is needed to enable multiplexed detection and real-time monitoring of antibiotics.

## Figures and Tables

**Table 1:** Antibiotic concentrations in waters.

Country/region	Category	Concentration (ng/mL)	Reference
Musi River, South India	Ciprofloxacin	6.59–5.53 × 10^3^	[28]
	Ofloxacin	1.55–318.1	
	Enrofloxacin	2.57–123.4	
	Norfloxacin	16.14–217.5	
	Pefloxacin	0.74–44.34	
	Difloxacin	0.47–37.74	
	Lomefloxacin	3.59–10.32	

Han River, South Korea	Oxytetracycline	1.236	[29]
	Tetracycline	2.093	
	Chlorotetracycline	0.793	

Wangyu River, China	Tetracyclines	1.082–15.310	[26]
	Quinolones	0.225–1.325	
	Sulfonamides	NA–0.888	
	Macrolides	0.012–0.017	

Msunduzi River, South Africa	Nalidixic acid	23.504	[30]
Vietnam	Sulfamethazine	19	[31]
Mozambique	Sulfamethoxazole	53.8	[32]
Kenya	Sulfamethoxazole	38.9	[33]
Croatia	Sulfamerazine	11	[34]

Lake Poyang, China	Sulfadiazine	5.62 × 10^−2^	[35]
	Oxytetracycline	4.87 × 10^−2^	
	Doxycycline	3.97 × 10^−2^	

**Table 2:** Summary of liquid chromatography-based conventional methods for the detection of antibiotics.

Conventional methods	Analytes	LOQ		LOD (ng/mL)	Reference
		Lower limit	Upper limit		
HPLC-RLS	Enoxacin	NA	NA	5.1	[67]
	Ofloxacin	NA	NA	3.1	
	Lomefloxacin	NA	NA	4.2	
	Gatifloxacin	NA	NA	3.8	
	Sparfloxacin	NA	NA	17.5	

HPLC-DAD	Sulfonamide	NA	NA	0.54∼3.67	[68]
LC-MS/MS	Macrolide	3.0 ng/L	40.0 ng/L	10^−3^∼1.5 × 10^−2^	[46]
HPLC-UV	Fluoroquinolones	NA	NA	0.3∼1.4	[69]
HPLC-FLD	Fluoroquinolones	NA	NA	1.4 × 10^−4^∼6.1 × 10^−4^	[70]

HPLC-RLS: high-performance liquid chromatography-tandem resonance light scattering; DAD: diode array detector; UV: ultraviolet detection; FLD: fluorescence detector; LC‐MS/MS: liquid chromatography with tandem mass spectrometry; LOQ: limit of quantification (upper and lower limits of the method are extremes of the linear range of the method); NA: not available.

**Table 3:** Summary of optical/electrochemical biosensors for the detection of antibiotics.

Biosensor	Sensor type	Bioreceptor	Analytes	LOQ (ng/mL)		LOD	Reference
				Lower limit	Upper limit		
Optical biosensors	Fluorescent biosensors	Aptamer	Ampicillin	0.1	100	0.07	[100]
		Aptamer	Oxytetracycline	25	1000	25	[85]
		Aptamer	Kanamycin	0.242	9.68	0.155	[101]
		Aptamer	Streptomycin	NA	NA	31.7	[102]
	Colorimetric biosensors	Aptamer	Kanamycin	9.7 × 10^−3^	2.42	4.85 × 10^−3^	[103]
		Enzyme	Oxytetracycline	23	4.6 × 10^2^	12	[78]
		Enzyme	Tetracycline	44.4	444	20	
		Enzyme	Doxycycline	23.1	462	22.2	
		Aptamer	Streptomycin	NA	NA	42.5	[104]
		Aptamer	Chloramphenicol	0.323	38.76	0.14	[105]
	Chemiluminescent biosensors	Aptamer	Kanamycin	NA	NA	0.03	[106]
		Aptamer	Streptomycin	NA	NA	0.33	
		Aptamer	Chloramphenicol	0.01	0.20	0.01	[107]
	Surface plasmon resonance biosensors	Antibody	Amoxicillin	0.1	2.0	0.022	[108]
		Antibody	Fluoroquinolone	NA	NA	0.07	[81]
		—	Erythromycin	4.99 × 10^3^	4.99 × 10^4^	290	[109]

Electrochemical biosensors	Amperometric biosensors	Aptamer	Kanamycin	NA	NA	4.23 × 10^−2^	[110]
		Aptamer	Streptomycin	NA	NA	2.62 × 10^−2^	
		Aptamer	Oxytetracycline	10	75	1.125	[111]
	Photoelectrochemical biosensors	Aptamer	Chloramphenicol	3.23 × 10^−4^	32.3	0.7 × 10^−4^	[112]
	Impedimetric biosensors	Aptamer	Penicillin	0.1	200	0.057	[113]
		Aptamer	Ampicillin	3.49 × 10^−2^	3.49 × 10^4^	NA	[114]
		Aptamer	Kanamycin A	4.84	4.84 × 10^6^	NA	
		Aptamer	Penicillin	0.1	200	0.057	[113]
		Antibody	Tetracycline	0.08	1.0	0.0321	[115]
		Antibody	Tetracycline	4.44 × 10^−6^	444	1.69 × 10^−6^	[116]
		Aptamer	Ampicillin	6.3	900	NA	[117]

**Table 4:** A general comparison of different methods used for antibiotic analysis.

Method	Advantage	Disadvantage	Reference
Conventional detection methods	(1) They can be used routinely to identify and quantify antibiotics (2) They have high selectivity and sensitivity	(1) Matrix effects lead to inaccurate results (2) Instruments and standard samples are expensive (3) They require complex pretreatment and long detection time (4) Skilled personnel are required to operate equipment and data	[77]

Biosensor methods	(1) The detection is fast and efficient (2) Detection does not require complex pretreatment (3) The method exhibits rapid detection	(1) The detection accuracy is not as high as the instrument (2) The initial development cost is high	[121]
